# Evaluation of urinary metabolites as biomarkers for occupational p-chloronitrobenzene exposure: a pilot study

**DOI:** 10.1038/s41598-025-96891-x

**Published:** 2025-04-15

**Authors:** Peng Wang, Lifang Han, Hua Zou, Yiyao Cao, Xiangjing Gao, Hong Ren, Qiuliang Xu

**Affiliations:** 1 Institute of Occupational Health and Radiation Protection, Zhejiang Provincial Center for Disease Control and Prevention, Hangzhou, China; 2 Department of Health Hazards Control, Shaoxing Shangyu District Center for Disease Control and Prevention, Shangyu, Shaoxing China

**Keywords:** Occupational exposure, p-Chloronitrobenzene, Biomarkers, Metabolites, Biomarkers, Analytical chemistry, Disease prevention, Occupational health, Public health

## Abstract

We explored the feasibility of using urinary metabolites of p-chloronitrobenzene (p-CNB) as exposure biomarkers of occupational p-CNB exposure. Forty-two workers exposed to p-CNB during their jobs at a chemical enterprise in Shaoxing, Zhejiang Province, China were included in the exposure group, while administrative personnel who do not come into contact with p-CNB at work were included in the control group. Each worker in the exposure group was equipped with a personal air sampler to collect airborne p-CNB samples, and urine samples were collected at the conclusion of each shift. After sample collection, the airborne p-CNB concentrations and urinary metabolite concentrations were detected by gas chromatography-tandem mass spectrometry and ultra-performance liquid chromatography - quadrupole - orbitrap high resolution mass spectrometry, respectively. The urinary metabolite concentrations were corrected by the content of urinary creatinine. Subsequently, the correlations between the urinary metabolite concentrations and the p-CNB time-weighted average (TWA) concentrations were assessed using correlation analysis. All p-CNB TWA concentrations measured in this study were below the occupational exposure limit in the Chinese national standards. In the exposure group, N-acetyl-S-(4-nitrophenyl)-L-cysteine (NANPC), 2-chloro-5-nitrophenol (2C5NP), p-chloroacetanilide (p-CAA), p-chlorooxanilic acid (p-COA), 2-amino-5-chlorophenol (2A5CP), and p-chloroaniline (p-CA) were detected at varying levels. The percentages of NANPC, 2C5NP, p - CAA, p - COA, 2A5CP, and p - CA were 64.1%, 5.1%, 0.3%, 15.1%, 5.1%, and 10.3%, respectively. We found extremely significant positive relationships (*p* < 0.01) between the urinary metabolite concentrations (p-CA, 2C5NP, 2A5CP, NANPC, and p-COA) and the p-CNB TWA concentrations, with respective correlation coefficients of 0.827, 0.673, 0.790, 0.714, and 0.741. Thus, these five metabolites may be used as exposure biomarkers of occupational p-CNB exposure. Moreover, among these metabolites, NANPC was identified as the most suitable exposure biomarker because it had the highest correlation coefficient and the highest content in urine.

**Supplementary Information:**

The online version contains supplementary material available at 10.1038/s41598-025-96891-x.

## Introduction

p-Chloronitrobenzene (p-CNB) is used primarily as a chemical intermediate in the production of rubber chemicals, lumber preservatives, dyes, drugs, corrosion inhibitors, pesticides, and photographic chemicals^1,2^. China is the leading producer and consumer of p-CNB worldwide^3,4^, suggesting that a significant portion of the population may be at risk of occupational exposure to this substance.

p-CNB has been shown to have irritant effects on both the mucous membrane and skin, potentially resulting in the development of methemoglobinemia^5,6^. Patients with acute p-CNB poisoning may exhibit symptoms such as cyanosis of the skin and mucous membranes, red blood cell rupture, and hemolysis caused by red blood cell rupture^6–8^. In addition, p-CNB and its metabolites can directly damage the liver and kidneys, and the release of hemoglobin following the rupture of red blood cells can also lead to liver and kidney damage^6–8^. The American Conference of Governmental Industrial Hygienists has confirmed the carcinogenic potential of p-CNB based on animal studies^9^, and the United States Environmental Protection Agency has classified p-CNB as a potential human carcinogen^10^. Therefore, occupational exposure to p-CNB may be associated with severe health risks.

Exposure assessment is widely recognized as an effective tool that can control occupational hazards of p-CNB exposure. Numerous countries have implemented occupational exposure limits and developed techniques for evaluating p-CNB in workplace air to assess worker exposure. While workplace air monitoring provides an initial evaluation of external exposure via respiratory inhalation, factors such as the use of personal protective equipment create challenges in accurately assessing exposure levels. Furthermore, p-CNB can be absorbed through dermal contact^11^; thus, workplace air monitoring may not provide an accurate assessment of the actual level of p-CNB exposure. In contrast, biological monitoring provides a comprehensive understanding of an individual actual exposure and effectively addresses the limitations of workplace air monitoring^12^.

The identification of biomarkers is essential prior to conducting a biological monitoring. Several studies have suggested the utility of urinary diazo-positive metabolites as biomarkers for p-CNB exposure^13–15^. Nevertheless, the specificity of urinary diazo-positive metabolites is poor, and exposure to other aromatic nitroamino compounds or certain medications (e.g., allergy drugs, antihypertensive drugs, and analgesics) can interfere with the results of p-CNB exposure assessments based on urinary diazo-positive metabolites^13–15^.

**Fig. 1:**
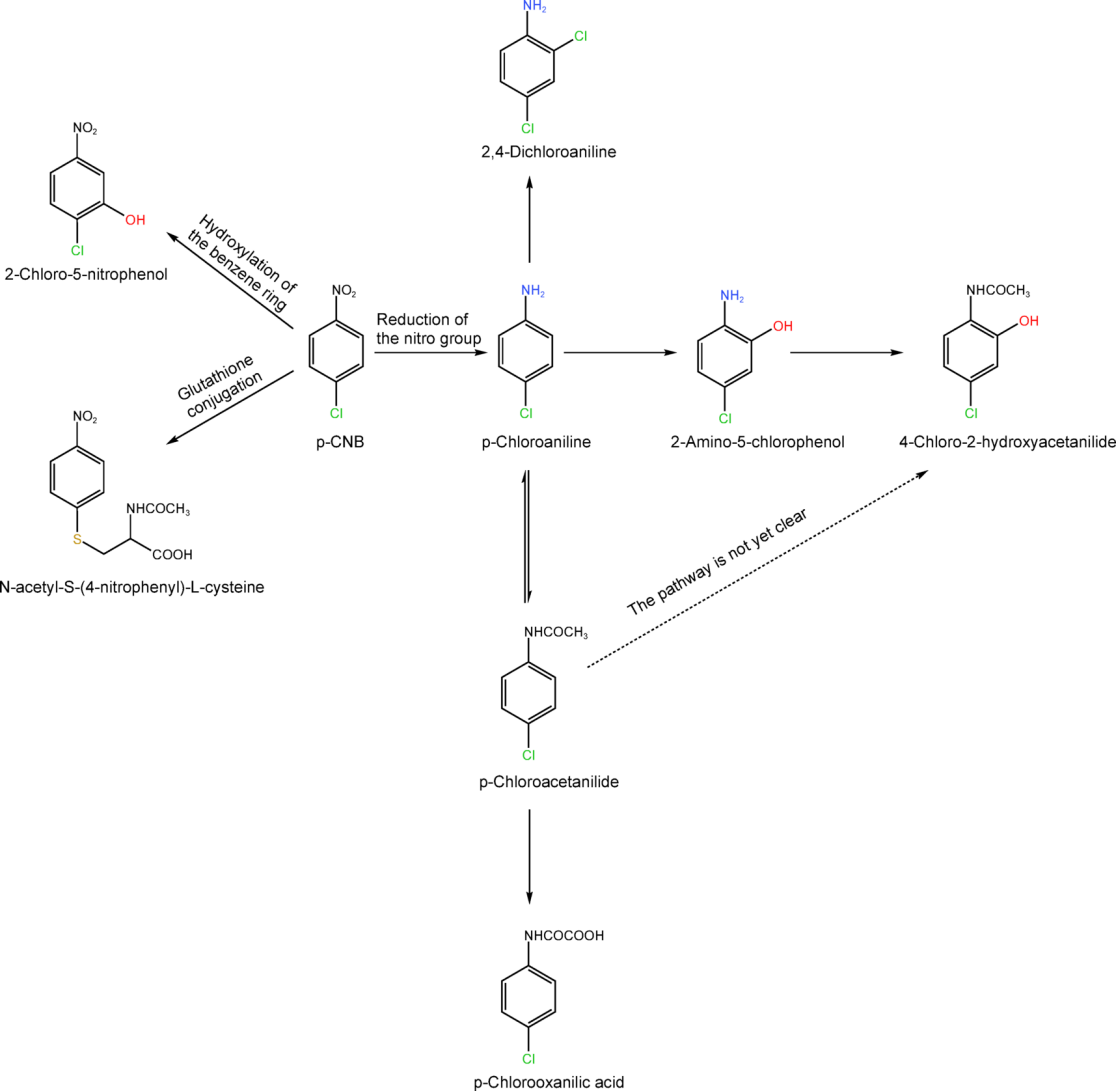
p-CNB metabolic processes.

To evaluate the toxicity of p-CNB on genes, the hemoglobin adduct for p-CNB was proposed as a biomarker; however, it reflects the cumulative exposure level of p-CNB over the past few weeks and cannot reflect the short - term exposure level^16^. Therefore, the identification of appropriate biomarkers is crucial for evaluating internal exposure to p-CNB. Exposure biomarkers are biomarkers that have been demonstrated to effectively indicate the overall body burden or total absorbed dose^17–19^. The identification of exposure biomarkers requires the study of metabolic processes in vivo. Animals studies have demonstrated that approximately two thirds of the administered dose of p-CNB is excreted through urine following metabolic processes^20^. Although unchanged p-CNB was not detected in the urine of individuals dosed with p-CNB, eight metabolites were identified: N-acetyl-S-(4-nitrophenyl)-L-cysteine (NANPC), 2-chloro-5-nitrophenol (2C5NP), p-chloroaniline (p-CA), p-chlorooxanilic acid (p-COA), 2-amino-5-chlorophenol (2A5CP), 4-chloro-2-hydroxyacetanilide (4C2HAA), 2,4-dichloroaniline (24DCA), and p-chloroacetanilide (p-CAA)^21,22^. In acutely poisoned individuals, the content of NANPC [the mean residence time (*MRT*) in humans was 7.0 d] was found to be the highest, and the levels of 2C5NP (MRT in humans = 6.7 d), p-CA (*MRT* in humans = 10.0 d), 2A5CP (*MRT* in humans = 6.0 d), and p-COA (*MRT* in humans not documented) were also relatively high; a small amounts of 24DCA (MRT in humans = 3.7 d) was detected, while only trace amounts of 4C2HAA and p-CAA (*MRT* in humans not documented) were found^21^. The corresponding metabolic processes are shown in Fig. 1. Although several scholars have examined the potential of urinary metabolites such as NANPC, p-CA, and 2C5NP as exposure biomarkers of p-CNB exposure, the correlations between these urinary metabolites of p-CNB and the airborne p-CNB concentration have not been sufficiently validated^23^.

Our objective was to identify urinary metabolites of p-CNB that could serve as exposure biomarkers, and to explore the impacts of common lifestyle habits such as smoking and drinking on these biomarkers. This was intended to provide appropriate biological monitoring indicators for evaluating the internal exposure to p-CNB. In this study, the proportion of workers who regularly smoked or drank was extremely low. As the limited data might not be representative, we disregarded the impacts of factors such as smoking and drinking and conducted a preliminary study solely from the perspective of the “dose - response” relationship.

## Materials and methods

### Study population

The exposure group consisted of 12 workers (11 males and 1 female) from the p-CNB production workshop and 30 workers (12 males and 18 females) from the p-CNB usage workshop of a chemical enterprise in Shaoxing, Zhejiang Province, China. The workers in the exposure group operate in a mobile fashion while wearing long-sleeved jackets, trousers, and rubber gloves; however, they do not wear protective masks. The workers operate in four 8-h shifts with three shift changes. The age range of the workers was 24 to 52 years with a median age of 38. The primary tasks in the p-CNB production workshop include nitrification and separation, whereas the main activities in the usage workshop include etherification, distillation, and p-nitrophenol sodium treatment. The control group comprised 40 administrative personnel (25 males and 15 females) with no history of exposure to p-CNB. All participants completed a questionnaire survey, provided a urine sample, and signed an informed consent form. The questionnaire covered various topics including working conditions, job responsibilities, and age. This study was carried out in compliance with the Declaration of Helsinki and authorized by Ethics Committee of Zhejiang Provincial Center for Disease Control and Prevention (ID: 2022-019-01).

### Instruments and reagents

GilAir Plus personal air sampler (0 ~ 2 L/min) provided by Gilian Company (USA); Gilibrator-2 flow calibrator provided by Gilian Company (USA); 8890-7000D gas chromatography-tandem mass spectrometry (GC-MS/MS) provided by Agilent Technologies (USA); ultra-performance liquid chromatography - quadrupole - orbitrap high resolution mass spectrometry (UPLC-Q-Orbitrap HRMS) system provided by Thermo Scientific (USA). The UPLC-Q-Orbitrap HRMS system includes a Vanquish ultra-performance liquid chromatograph and an Orbitrap Exploris 120 mass spectrometer.

NANPC (standard, > 98%) was from Tokyo Chemical Industry Co., Ltd.; p-CAA (standard, ≥ 99%), p-CA (standard, ≥ 99%), 24DCA (standard, ≥ 99%), and 2C5NP (standard, ≥ 98%) were from Aladdin Reagent (Shanghai) Co., Ltd.; 2A5CP (standard, > 98%) was from Shanghai Macklin Biochemical Co., Ltd.; p-COA (standard, 99%) and 4C2HAA (standard, 99%) were from Shandong Lvdedi Drug Research and Development Co., Ltd. The concentrated hydrochloric acid (36.0 − 38.0%) was from Sinopharm Chemical Reagent Co., Ltd. (China); the ammonium hydroxide (for LC-MS, ≥ 25%) was from Aladdin Reagent (Shanghai) Co., Ltd. The HPLC-grade solvents of acetonitrile, benzene, and methanol were from Fisher Scientific (USA); the HPLC-grade solvent of formic acid was from Shanghai Macklin Biochemical Co., Ltd. Solid phase extraction columns (Oasis MCX and HLB; 60 mg, 3 mL) were from Waters (USA).

### Air determination

The airborne p - CNB was collected using a silica gel tube or fiber filter paper. After solvent desorption, it was detected by GC-MS/MS.The specific procedures of the method are described in detail in the supplementary material. The precision, accuracy, limit of detection and limit of quantification of the method are listed in Table S1. In the exposure group, each worker was equipped with a personal air sampler to collect airborne p-CNB samples in November, which is in winter. Solvent desorption-type silicone tubes (front-end silicone/rear-end silicone 200 mg/100 mg) were used to collect p-CNB in the vapor state with a sampling flow rate of 50 mL/min. Glass fiber filter papers were used to collect p-CNB aerosols with a sampling flow rate of 1 L/min. The duration of the sampling period was 3 h. A calibrator was used to calibrate the sampling flow before and after sampling. The ultimate sampling flow rate was determined as the mean of the pre - and post - sampling flow rates. If the flow rate deviated by more than 5%, the corresponding sample was deemed invalid and excluded from analysis. Following sampling, the samples were sealed and transported to the laboratory in a clean container for analysis by GC-MS/MS. The airborne p-CNB concentration was determined by summing the aerosol and vapor p-CNB concentrations. The time-weighted average (TWA) concentration for each worker was obtained from the airborne p-CNB concentration.

### Urine collection and preservation

Following the collection of personal air samples for the workers, urine samples (minimum volume of 30 mL) were collected in polyethylene plastic cups with lids at the conclusion of the shift. The urine samples were stored in an ice box and immediately transported to the laboratory for analysis. If the samples could not be analyzed immediately, they were stored at − 80 °C.

### Determination of metabolites in urine

The conjugated forms of the p-CNB metabolites in the urine samples were concentrated and purified by solid-phase extraction after acid hydrolysis. The free forms of the metabolites were directly concentrated and purified by solid-phase extraction without hydrolysis. The metabolites were then detected by UPLC-Q-Orbitrap HRMS. The specific determination processes are described in our previous report^24^. The precision, accuracy, limit of detection and limit of quantification of the method are listed in Table S2. The metabolite concentrations were corrected using the creatinine concentration, which was detected in accordance with the Chinese national standard^25^.

### Statistical analysis

Statistical analysis was performed using SPSS 30 software. The Kolmogorov–Smirnov test was used to assess the normality of determined p-CNB concentrations in the air samples, the determined concentrations of p-CNB metabolites in urine samples, and worker age and gender with *p* > 0.05 indicating a normal distribution. Data conforming to a normal distribution were described as the mean ± standard deviation, and the pairwise difference was evaluated by a variance test with *p* < 0.05 indicating a significant difference. Data deviating from a normal distribution were described using the median and interquartile range (25th to 75th percentile), and the pairwise difference was assessed using the Mann–Whitney *U* test with *p* < 0.05 indicating a significant difference. Pearson correlation analysis was used to assess the correlations between pairs of variables with normal distributions, while linear regression was used to evaluate the relationships between variables, with *p* < 0.05 indicating a significant correlation. Spearman correlation analysis was used to assess the correlations between pairs of variables with non - normal distributions, with *p* < 0.05 indicating a significant correlation.

## Results

### The p-CNB TWA concentrations of workers

In the personal air samples collected from workers, p-CNB was present in the vapor form, as evidenced by the absence of p-CNB aerosols detected in samples collected using glass fiber filter papers. The general characteristics and p-CNB TWA concentrations of the workers are shown in Table 1. The p-CNB TWA concentrations of workers in the exposure group were all above the limit of quantification, with a median concentration of 1.39 µg/m^3^. The median p-CNB TWA concentration of workers in the production workshop was 24.48 µg/m^3^_,_ while that of workers in the usage workshop was 0.59 µg/m^3^. All p-CNB TWA concentrations measured in this study were below the occupational exposure limit in the Chinese national standards^11^. Kolmogorov–Smirnov test indicated normal distributions for participant age and gender, whereas the p-CNB TWA concentration data did not conform to a normal distribution. Mann–Whitney *U* test indicated a statistically significant difference in the p-CNB TWA concentrations between workers in the production workshop and the usage workshop (*p* < 0.01). Based on the p-CNB TWA concentrations, workers from the p-CNB production workshop were categorized as the high exposure group, while workers in the usage workshop were categorized as the low exposure group. Variance tests indicated that the worker ages and gender distributions were not significantly different between the exposure and control groups (*p* > 0.05).

**Table 1:** Characteristics and p-CNB TWA concentrations of workers in this study.

Group	Cases	Year	Gender man/woman	*p*-CNB TWA concentration (µg/m^3^)
				Median (25th–75th percentile)
Low-exposure group (usage workshop)	30	38.8 ± 6.6	12/18	0.59 (0.18–1.57)
High-exposure group (production workshop)	12	38.4 ± 8.4	11/1	24.48 (19.06–38.18)^a^
Exposure group	42	38.7 ± 7.0^b^	23/19^c^	1.39 (0.44–14.53)
Control group	40	37.9 ± 8.2	25/15	

Note: Compared to low-control group, ^a^*p* < 0.01; Compared to control group, ^b^*p >* 0.05, ^c^*p >* 0.05.

### The urinary metabolite concentrations of workers

Table 2 shows the urinary metabolite concentrations of workers. In the exposure group, 4C2HAA and 24DCA were not detected in any of the urine samples, whereas the remaining six metabolites were detected at varying levels. Notably, NANPC and p-CA were both detected in 100% of the urine samples. The detection rates of the six metabolites in the high - exposure group were all above 90% (91.5% for 2C5NP, and 100% for the others). Given that these detection rates were much higher than those in the low - exposure group, we chose the median data of the high - exposure group to calculate the ratios among the concentrations of various metabolites. The results showed that the percentages of NANPC, 2C5NP, p-CAA, p-COA, 2A5CP, and p-CA were 64.1%, 5.1%, 0.3%, 15.1%, 5.1%, and 10.3%, respectively. NANPC accounted for the vast majority of the total metabolites, while the content of p-CAA was the lowest (< 1%). The percentage levels of p-COA and p-CA were comparable, as were those of 2C5NP and 2A5CP. Moreover, the percentages of p-COA and p-CA were higher than those of 2C5NP and 2A5CP. These findings were generally consistent with the results of previous studies^21,22^. Kolmogorov–Smirnov test indicated that the concentrations of the above five metabolites(NANPC, 2A5CP, p-COA, p-CA, and 2C5NP) with relatively high contents did not conform to a normal distribution. Mann–Whitney *U* test showed that the five urinary metabolite concentrations were significantly higher in the high exposure group than in the low exposure group (*p* < 0.05). In addition, there were also significant correlations among the five metabolites (*p* < 0.01), and all the correlation coefficients were greater than 0.7. The specific data are shown in Table 3. No p-CNB metabolites were detected in the urine samples from the control group.

**Table 2:** Concentrations of metabolites in the urine samples of exposed workers.

Compound	High-exposure group			Low-exposure group			Control group
	Range (µg/g Cre)	Median (25th–75th percentile) (µg/g Cre)	Detection rate (%)	Range (µg/g Cre)	Median (25th–75th percentile) (µg/g Cre)	Detection rate (%)	Range (µg/g Cre)
NANPC	221.6–1627.3	425.5(363.4, 724.6)^a^	100	3.3–152.4	36.2(20.3, 59.5)	100	ND
2C5NP	ND − 209.4	34.1(20.4, 62.1)^b^	91.7	ND–19.4	/	23.3	ND
p-CAA	ND − 5.2	1.6(1.3, 3.9)	100	ND–1.6	/	10.0	ND
p-COA	60.1–325.3	100.2(68.7, 156.8)^c^	100	ND–28.0	7.7(5.1, 13.3)	90.0	ND
4C2HAA	ND	/	0	ND	/	0	ND
2A5CP	3.8- 130.4	34.1(12.6, 56.1)^d^	100	ND–22.2	/	46.7	ND
p-CA	14.2–203.4	68.4(28.6, 100.5)^e^	100	4.0–28.9	10.9(6.3, 13.8)	100	ND
24DCA	ND	/	0	ND	/	0	ND

Notes: ND means a metabolite was below the limit of detection. Compared to the low-exposure group: ^a^*p* < 0.05, ^b^*p* < 0.05, ^c^*p* < 0.05, ^d^*p* < 0.05, ^e^*p* < 0.05.

**Table 3:** Statistical analysis of the correlations between the metabolites.

Group	Correlation coefficient (*R*)	*p*-values
NANPC and p-COA	0.834	< 0.01
NANPC and p-CA	0.810	< 0.01
NANPC and 2C5NP	0.908	< 0.01
NANPC and 2A5CP	0.803	< 0.01
p-COA and p-CA	0.912	< 0.01
p-COA and 2C5NP	0.808	< 0.01
p-COA and 2A5CP	0.794	< 0.01
p-CA and 2C5NP	0.825	< 0.01
p-CA and 2A5CP	0.909	< 0.01
2C5NP and 2A5CP	0.719	< 0.01

### Correlations between the urinary metabolite concentrations and the p-CNB TWA concentration

**Fig. 2:**
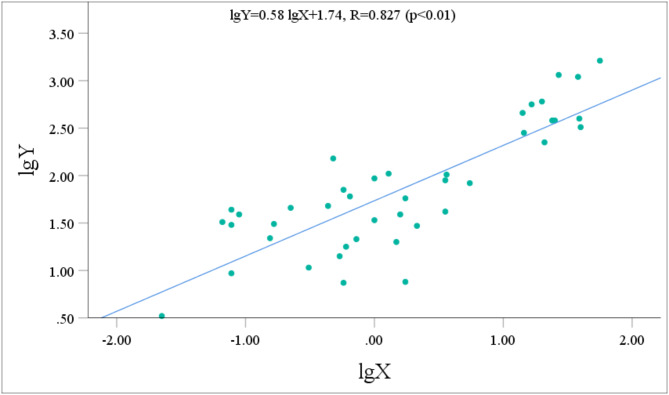
Correlation of urinary NANPC concentration (Y, in µg/g Cre) with the p-CNB TWA concentration (X, in µg/m^3^) in 42 occupational p-CNB-exposed workers.

**Fig. 3:**
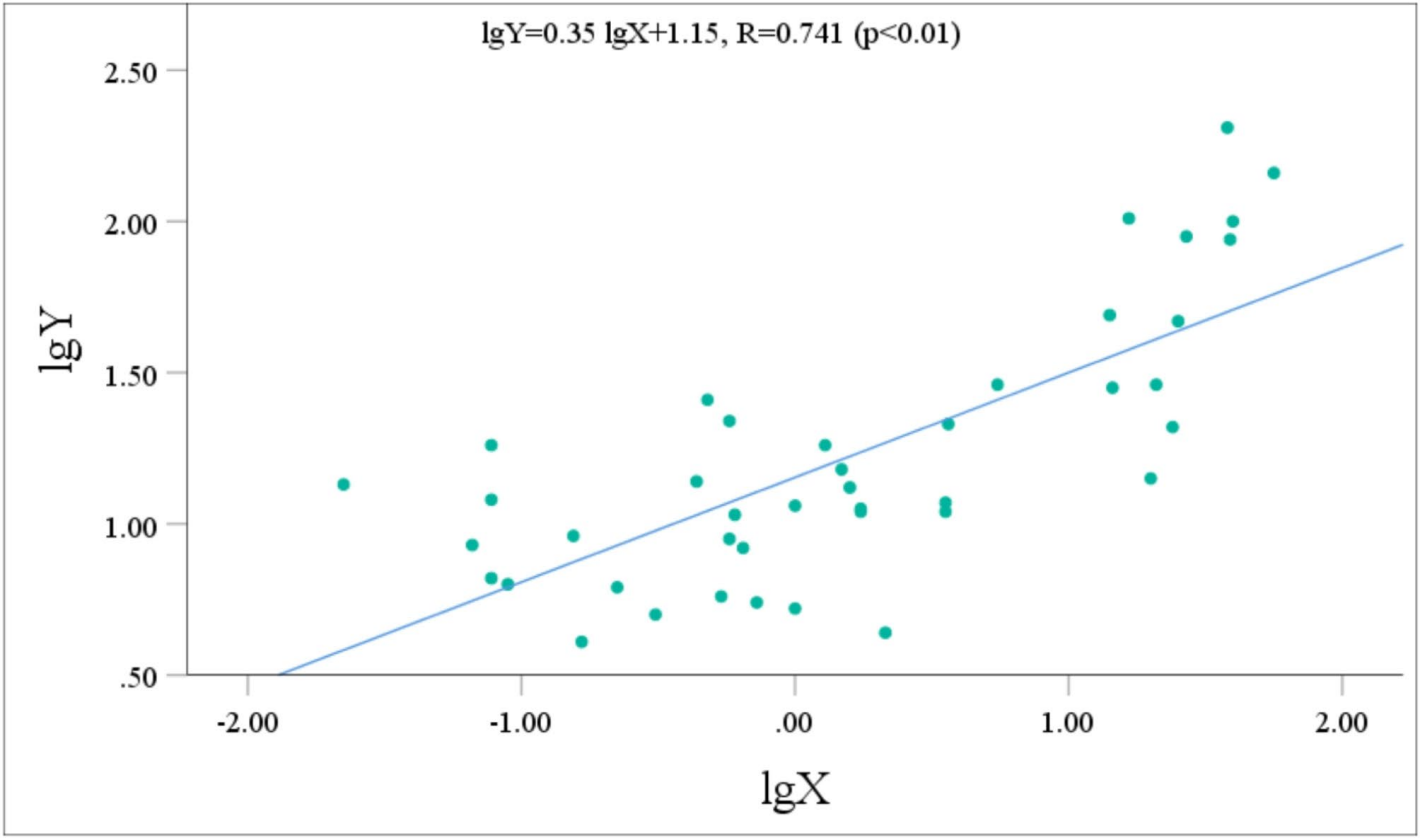
Correlation of urinary p-CA concentration (Y, in µg/g Cre) with the p-CNB TWA concentration (X, in µg/m^3^) in 42 occupational p-CNB-exposed workers.

**Fig. 4:**
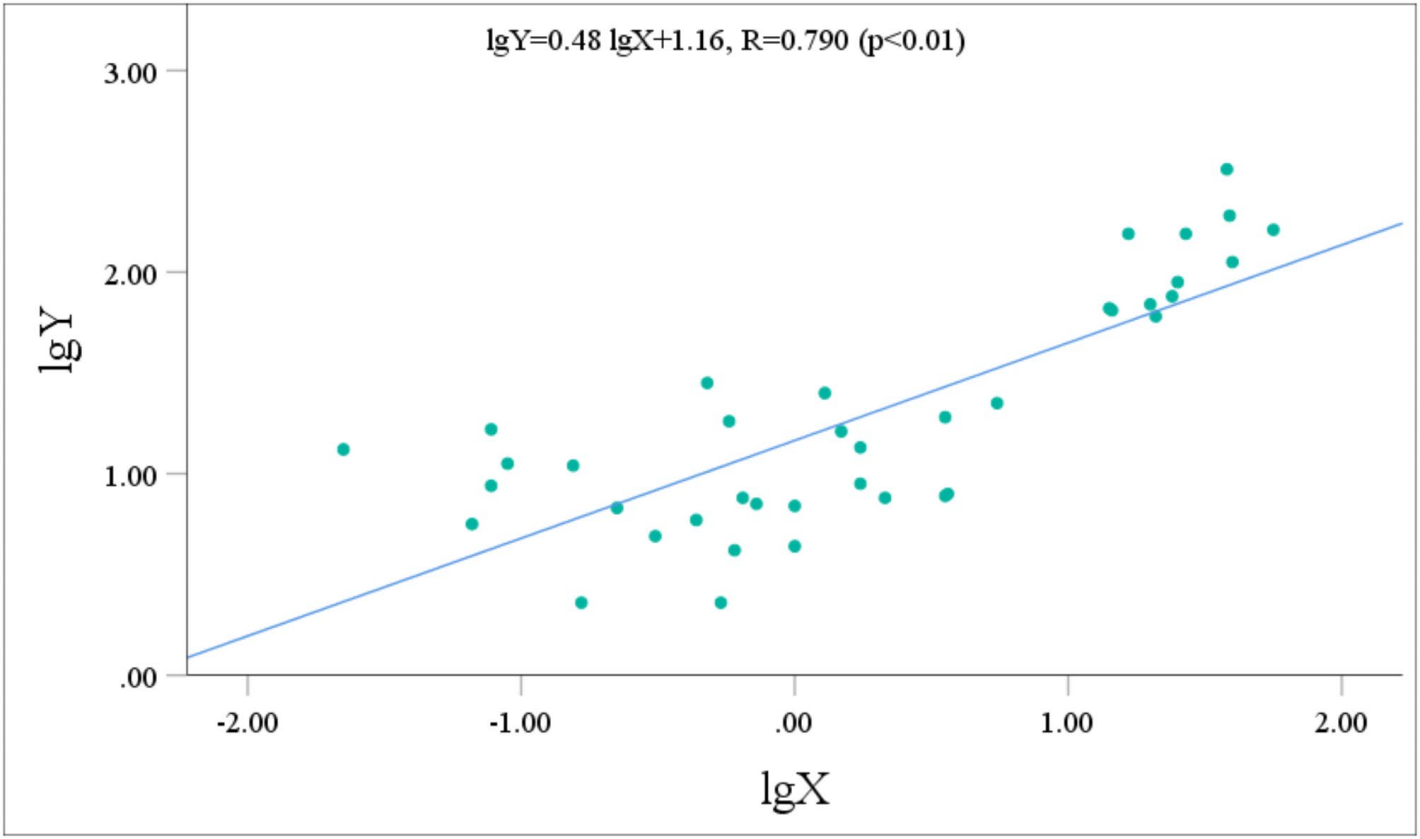
Correlation of urinary p-COA concentration (Y, in µg/g Cre) with the p-CNB TWA concentration (X, in µg/m^3^) in 39 occupational p-CNB-exposed workers.

**Fig. 5:**
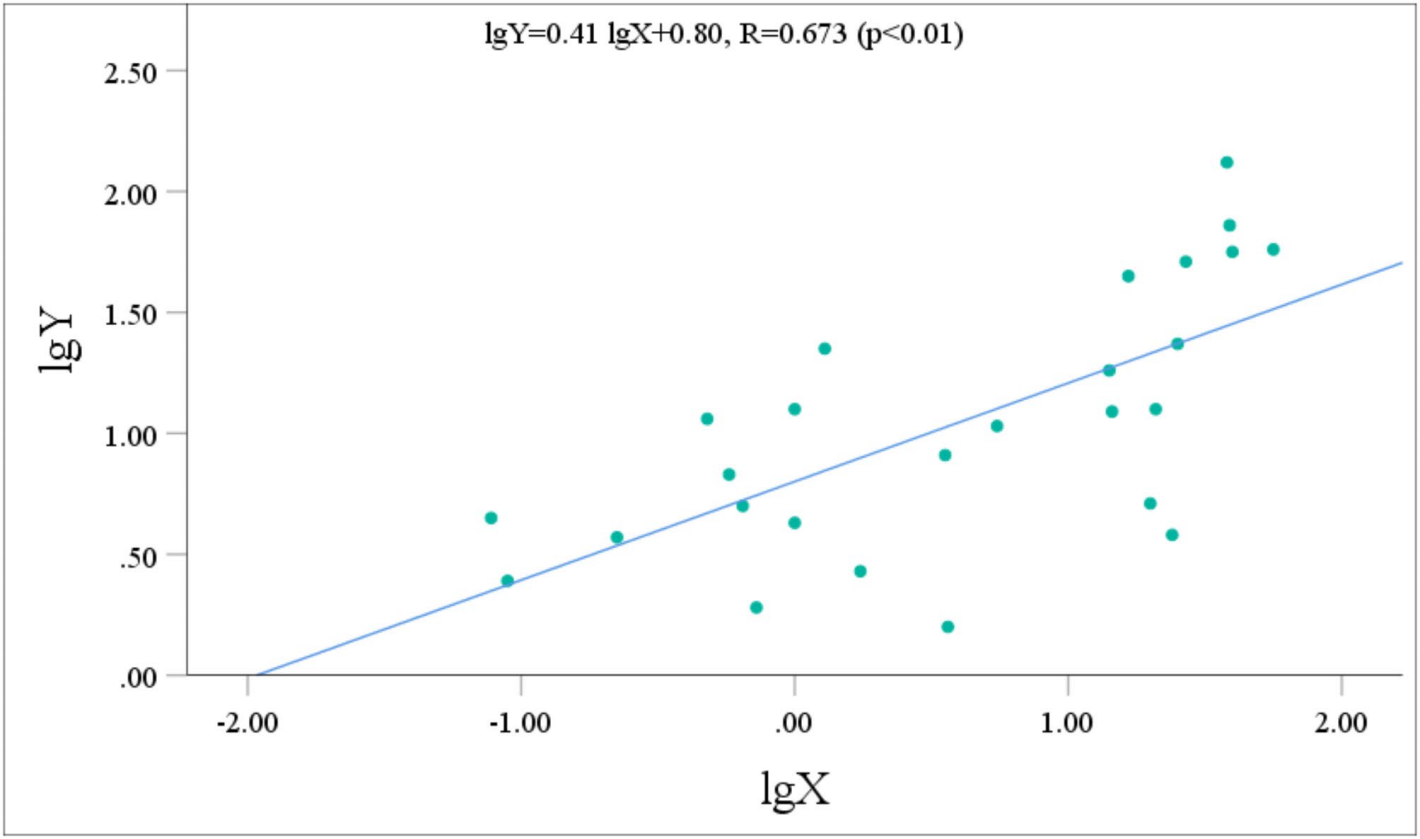
Correlation of urinary 2A5CP concentration (Y, in µg/g Cre) with the p-CNB TWA concentration (X, in µg/m^3^) in 26 occupational p-CNB-exposed workers.

**Fig. 6:**
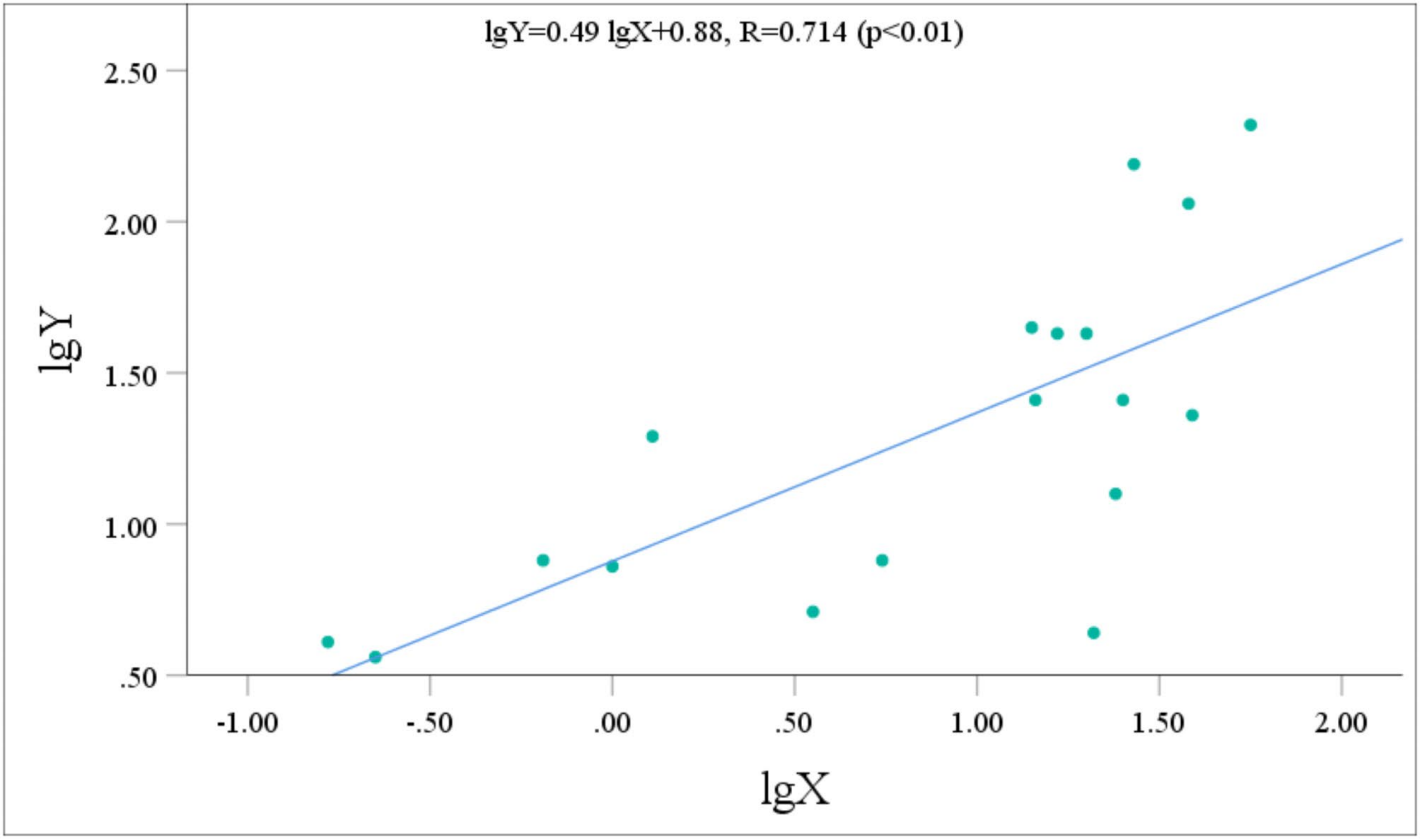
Correlation of urinary 2C5NP concentration (Y, in µg/g Cre) with the p-CNB TWA concentration (X, in µg/m^3^) in 18 occupational p-CNB-exposed workers.

The urinary metabolite concentrations and the p-CNB TWA concentrations all followed normal distributions after logarithmic transformation. Pearson correlation analysis was performed to assess the relationship between the concentrations of five metabolites with relatively high contents (NANPC, 2A5CP, p-COA, p-CA, and 2C5NP) and the p-CNB TWA concentration. Prior to performing statistical analysis, the data points that fell below the limit of detection were removed. After the exclusion of these data points, the statistical analysis included 42 workers for NANPC and p-CA, 26 workers for 2A5CP, 39 workers for p-COA, and 18 workers for 2C5NP. The analysis revealed that the urinary concentrations of NANPC, 2A5CP, p-COA, p-CA, and 2C5NP were extremely positively correlated (*p* < 0.01) with the p-CNB TWA concentration (Figs. 2, 3, 4, 5 and 6). The correlation coefficient between the urinary NANPC concentration and p-CNB TWA concentration reached 0.827, indicating a strong positive correlation. The urinary concentrations of 2A5CP, p-COA, 2C5NP, and p-CA also showed comparatively strong positive correlations with the p-CNB TWA concentration, with correlation coefficients ranging from 0.673 to 0.790. The data of the p - CNB TWA concentrations and the urinary concentrations of the five metabolites without logarithmic transformation were also evaluated by Spearman correlation analysis, and all showed significant correlations (*p* < 0.01), with correlation coefficients all greater than 0.67 (Table 4).

**Table 4:** Statistical analysis of the correlations between the concentrations of metabolites without logarithmic transformation and the p-CNB TWA concentrations without logarithmic transformation.

Compound	Correlation coefficient (*R*)	*p*-values
NANPC	0.752	< 0.01
p-COA	0.737	< 0.01
p-CA	0.679	< 0.01
2A5CP	0.697	< 0.01
2C5NP	0.725	< 0.01

## **Discussion**

Environmental monitoring can obtain the concentration, distribution and variation patterns of harmful substances, which helps to identify the sources and transmission routes of these substances. However, it cannot accurately reflect the actual exposure and absorption doses of different individuals. In contrast, biological monitoring can not only directly reflect the total exposure of an individual through routes such as respiration, dermal contact and ingestion, demonstrating individual differences, but also, to some extent, reflect the body’s absorption, metabolism and accumulation of harmful substances. Moreover, it can detect low - dose exposures that are difficult to find by environmental monitoring. Nevertheless, factors like an individual’s physiological state and metabolic capacity can cause significant variability in the results of biological monitoring^26^. Additionally, there is a lack of biological monitoring indicators for many harmful substances. The focus of this study is to find exposure biomarkers suitable for the assessment of p-CNB. Compared with broad biomarkers, exposure biomarkers are more targeted. They can promptly reflect recent exposures, which is conducive to tracking sudden exposure incidents.

p-CNB can be assimilated into the human body via the respiratory tract and through dermal contact. Therefore, the external exposure of workers to p-CNB includes the inhaled dose from the respiratory tract and the absorbed dose from dermal contact. In this study, the sampling was conducted in winter, when the workers were wearing long-sleeved jackets, trousers, and rubber gloves. Thus, the dose absorbed through dermal contact may be negligible, and the p-CNB TWA concentrations of the workers could reflect their true level of external exposure. In this study, although the air monitoring duration was 3 h and did not cover the entire work shift, these 3 h fully encompassed all the workers’ tasks. During the remaining time periods, the workers basically repeated these tasks. In addition, the workers adopted standardized operations with a stable working method. Therefore, the 3 - hour air monitoring can fully reflect the exposure situation throughout the work shift.

Based on existing studies^21^, the *MRTs* of NANPC, p-CA, 2C5NP, and 2A5CP in humans are 7.0, 10.0, 6.7, and 6.0 d, respectively. However, the *MRTs* of these metabolites were derived from patients suffering from acute poisoning, who were distinguished by their exposure to a substantial quantity of p-CNB. Animal experimental investigations have demonstrated that the *MRT* of every metabolite rises in tandem with the increase in the p-CNB dose^20^. When p-CNB was administered to rats at a dosage of 30 mg/kg, the *MRTs* of 2C5NP, NANPC, p-CA, and 2A5CP were measured to be 21.5 h, 18.5 h, 25.4 h, and 21.0 h respectively. Consequently, the half - lives (calculated as *t*_1/2_=ln2×*MRT*) of 2C5NP, NANPC, p-CA, and 2A5CP can be computed to be 14.9 h, 12.8 h, 17.6 h, and 14.6 h respectively. In reality, during normal operations, workers do not come into contact with such a high dose of p-CNB. As a result, the half - lives of these metabolites in the bodies of workers during normal operations are highly likely to fall within the range of several hours to about a dozen hours. Moreover, certain researchers collected the pre - shift urine of workers during normal operations, yet these metabolites were not detected in the samples^23^. This clearly indicates that following the termination of p-CNB exposure and prior to the next shift, these metabolites had been eliminated to a level beneath the limit of quantification of the detection method, suggesting that there is no accumulation during the workweek.

However, there is a lack of published studies on the mean residence time of p-COA in humans. p-COA is a conjugated form of p-CA that can be converted into p-CA through hydrolysis^21^. Based on the molecular weights of p-CA and p-COA, the quantity of p-CA generated following the complete hydrolysis of p-COA is approximately 0.64 times the initial quantity of p-COA. The conjugated forms of p-CA also include p-CAA, glucuronate, and sulfate^21,23^. In this study, the concentrations of p-CA hydrolyzed from p-COA (calculated by multiplying the p-COA concentration by 0.64) were 64.1 (44.0, 100.3) µg/g Cre and 4.9 (3.3, 8.5) µg/g Cre in the high and low exposure groups, respectively. These forms constituted most of the total p-CA concentration, particularly in the high exposure group. Therefore, we concluded that p-COA is the primary conjugated form of p-CA. In light of this discovery, we can plausibly infer that the mean residence time of p-COA in humans is comparable to that of p-CA.

A valid biomarker must provide a requisite level of specificity and sufficient sensitivity^26^. To exhibit “sufficient sensitivity,” the biomarker must demonstrate a dose-response correlation with the external exposure level, and this relationship must be maintained even at low exposure levels.

The objective of this study was to determine whether any of the eight urinary metabolites of p-CNB can serve as exposure biomarkers with superior specificity compared to diazo-positive metabolites. Compared with the control group, the urinary concentrations of six metabolites (NANPC, 2A5CP, p-COA, p-CA, 2C5NP, and p-CAA) were elevated to varying extents in the exposure group. These findings indicate that exposure to p-CNB induced alterations in urinary metabolite levels among the exposed workers. In addition, all the above metabolites, with the exception of p-CAA in the exposure group, showed significant elevations compared with the control group, with NANPC showing the greatest elevation. These findings suggest that NANPC, 2A5CP, p-COA, p-CA, and 2C5NP may serve as potential exposure biomarkers of occupational p-CNB exposure.

Pearson correlation analysis was further employed to verify the feasibility of the five metabolites as exposure biomarkers of p-CNB. The findings revealed an extremely significant strong positive relationship between the p-CNB TWA concentration and the urinary concentrations of NANPC (*p* < 0.01, *R* = 0.823), as well as extremely significant comparatively strong positive correlations between the p-CNB TWA concentrations and the urinary concentrations of 2A5CP, p-COA, 2C5NP, and p-CA (*p* < 0.01, *R* = 0.673 ~ 0.790). Furthermore, the p-CNB TWA concentrations detected for all workers in our study were below one-tenth of the limit for occupational exposure (0.6 mg/m^3^)^11^; thus, we believe that the detected concentrations of p-CNB were at a low level. At these low exposure levels, we found dose-response relationships between the p-CNB TWA concentration and the urinary concentrations of these five metabolites (NANPC, 2A5CP, p-COA, 2C5NP, and p-CA), suggesting that these metabolites exhibit sufficient sensitivity as exposure biomarkers of p-CNB exposure. In addition, there was a strong correlation among these five metabolites. Moreover, there was also a significant correlation between the concentrations of these five metabolites without logarithmic transformation and the p - CNB TWA concentrations without logarithmic transformation. This further demonstrates that these five metabolites are suitable as markers. Among these metabolites, NANPC was identified as the most suitable exposure biomarker because it had the highest correlation coefficient and the highest content in urine.

## **Conclusions**

We conducted a systematic study of potential biomarkers for p-CNB exposure. The results revealed extremely significant positive correlations between the external exposure levels of p-CNB workers and the urinary levels of five metabolites: NANPC, 2A5CP, p-COA, 2C5NP, and p-CA. Thus, these metabolites show promise as exposure biomarkers of occupational p-CNB exposure, among which NANPC is the most suitable exposure biomarker. Our findings provide insights into biological monitoring indicators for p-CNB.

It should be noted that the effects of factors such as smoking and alcohol consumption were not considered in this study. Future research should explore the influences of these factors.

## Electronic supplementary material

Below is the link to the electronic supplementary material.

Supplementary Material 1

## Data Availability

Data is provided within the manuscript or supplementary information files.
