# Supplementary material for: Evaluation of urinary metabolites as biomarkers for occupational p-chloronitrobenzene exposure: a pilot study
Source: Sci Rep. 2025 Apr 15;15:13011. doi: 10.1038/s41598-025-96891-x (PMC12000600; doi:10.1038/s41598-025-96891-x)
Supplement: Supplementary file 1 — Supplementary Material 1 [file 41598_2025_96891_MOESM1_ESM.docx]

**Supplementary Material for**

**Evaluation of Urinary Metabolites as Biomarkers for Occupational p-Chloronitrobenzene Exposure: A Pilot Study**

Peng Wang^1†^, Lifang Han^2†^, Hua Zou^1^, Yiyao Cao^1^, Xiangjing Gao^1^, Hong Ren^1^, Qiuliang Xu^1*^

^1^Institute of Occupational Health and Radiation Protection, Zhejiang Provincial Center for Disease Control and Prevention, Hangzhou, China

^2^ Department of Health hazards control, Shaoxing Shangyu District Center for Disease Control and Prevention, Shangyu, Shaoxing, China.

*Corresponding author: Qiuliang Xu, E-mail address:[qlxu@cdc.zj.cn](mailto:hzou@cdc.zj.cn).

†These authors have contributed equally to this work

**Summary**

This supplementary material includes detailed description of analytical procedures for determination of p-CNB in air, as well as 2 tables demonstrating precision, accuracy, limit of detection and limit of quantification of air determination method(Table S1) and urinary metabolites determination method(Table S2).

**The method of air determination**

**1. Sample collection**

(1) Vapor - state p-CNB

Solvent - desorbing silica gel tubes were used for collection. At the sampling location, both ends of the solvent - desorbing silica gel tube were opened, and air samples were collected at a flow rate of 50 mL/min for 3 hours. After sampling, both ends of the silica gel tube were sealed.

(2) Aerosol - state p-CNB

Ultrafine glass fiber filter papers were used for collection. Air samples were collected at a flow rate of 1 L/min for 3 hours. After sampling, the sampling clip was opened, and the filter paper was taken out. The filter paper was folded in half with the dust - collecting side facing inwards and placed into a solvent desorption bottle, then transported and stored in a clean container.

**2. Sample processing**

(1) Silica gel tube sample

The front and back sections of the silica gel were transferred into desorption bottles, and 1.0 mL of methanol - toluene solution (5:95, V/V) was added to each bottle for

30-minute desorption.

(2) Ultrafine glass fiber filter paper sample

2.0 mL of methanol - toluene solution (5:95, V/V) was added to the desorption bottle containing the ultrafine glass fiber filter paper for 30-minute elution.

**3. Instrument conditions**

(1) Chromatographic conditions

Column: DB - 5MS (30 m × 0.25 mm × 0.25 µm); oventemperature program: nitial column temperature is 50 ℃, increased to 190 ℃ at a rate of 10 ℃/min; injector temperature: 250 ℃, injection in splitless mode; carrier gas(He) flow rate: 1.2 mL/min; injection volume: 1 µL.

(2) Mass spectrometric conditions

Transfer line temperature: 250 ℃; EI ion source, temperature: 230 ℃, ionization energy: 70 eV; quadrupole temperature: 150 ℃; solvent delay: 4.0 min; quenching gas (He) flow rate: 2.25 mL/min; collision gas (N_2_) flow rate: 1.5 mL/min; Multiple Reaction Monitoring (MRM) mode (quantitative ion pair: 156.9 > 99.0, collision energy: 15 eV; qualitative ion pair: 156.9 > 127.0, collision energy: 3 eV).

**Table S1.** Precision, accuracy, limit of detection and limit of quantification of the method

| Sampling medium | Limit of detection  (ng) | Minimum detectable concentration  (μg/m^3^) | Limit of quantification  (ng) | Minimum quantifiable concentration  (μg/m^3^) | Spiked  content  (ng) | Accuracy  (%) | CV  (%, n = 6) |
| --- | --- | --- | --- | --- | --- | --- | --- |
| Solvent desorption-type  silicone tube | 0.2 | 0.02 | 0.6 | 0.07 | 10 | 95.3 | 3.2 |
|  |  |  |  |  | 350 | 92.1 | 1.7 |
|  |  |  |  |  | 750 | 98.7 | 0.4 |
| Glass fiber filter paper | 0.4 | 0.04 | 1.2 | 0.13 | 20 | 92.1 | 3.3 |
|  |  |  |  |  | 700 | 93.1 | 2.5 |
|  |  |  |  |  | 1500 | 99.3 | 0.5 |

**Note:** The volumes of the desorption solutions for the solvent desorption-type silicone tubes and glass fiber filter papers are 1 mL and 2 mL respectively. The minimum detectable concentration and the minimum quantifiable concentration are calculated based on the collection of 9 L of air.

**Table S2.** Precision, accuracy, limit of detection and limit of quantification of the method

| Compound | Limit of detection  (μg/L) | Limit of quantification  (μg/L) | Spiked  concentration  (μg/L) | Intra-day | | Inter-day | |
| --- | --- | --- | --- | --- | --- | --- | --- |
|  |  |  |  | Accuracy  (%) | CV  (%, n = 6) | Accuracy  (%) | CV  (%, n = 18) |
| N-acetyl-S-(4-nitrophenyl)-L-cysteine | 0.8 | 2.6 | 15 | 89.5 | 2.7 | 88.8 | 3.7 |
|  |  |  | 100 | 87.3 | 2.2 | 86.8 | 2.6 |
|  |  |  | 300 | 86.4 | 3.2 | 86.5 | 3.0 |
| 2-Chloro-5-  nitrophenol | 2.7 | 8.9 | 15 | 83.3 | 6.6 | 82.4 | 8.5 |
|  |  |  | 100 | 86.2 | 3.8 | 86.3 | 4.2 |
|  |  |  | 300 | 85.5 | 4.0 | 86.1 | 4.9 |
| p-Chloroacetanilide | 0.2 | 0.6 | 15 | 99.7 | 1.5 | 98.4 | 2.1 |
|  |  |  | 100 | 98.3 | 0.4 | 97.9 | 1.0 |
|  |  |  | 300 | 96.6 | 1.4 | 98.4 | 2.1 |
| p-Chlorooxanilic acid | 1.9 | 6.3 | 15 | 76.0 | 3.2 | 78.5 | 3.5 |
|  |  |  | 100 | 76.1 | 1.0 | 77.4 | 4.3 |
|  |  |  | 300 | 76.5 | 3.6 | 76.1 | 4.8 |
| 4-Chloro-2-  hydroxyacetanilide | 0.5 | 1.7 | 20 | 100.7 | 1.4 | 102.1 | 2.5 |
|  |  |  | 150 | 102.3 | 0.9 | 102.9 | 1.3 |
|  |  |  | 400 | 100.4 | 1.3 | 99.8 | 1.6 |
| 2-Amino-5-chlorophenol | 0.9 | 3.1 | 20 | 82.1 | 4.4 | 83.9 | 4.4 |
|  |  |  | 150 | 87.6 | 7.0 | 84.9 | 7.8 |
|  |  |  | 400 | 96.0 | 5.6 | 95.2 | 4.9 |
| p-Chloroaniline | 1.0 | 3.3 | 20 | 82.8 | 2.1 | 84.5 | 3.5 |
|  |  |  | 150 | 85.7 | 5.4 | 87.4 | 4.6 |
|  |  |  | 400 | 85.7 | 5.0 | 86.2 | 5.8 |
| 2,4-Dichloroaniline | 7.7 | 25.6 | 100 | 80.1 | 5.1 | 81.6 | 5.9 |
|  |  |  | 350 | 82.3 | 1.3 | 84.3 | 3.3 |
|  |  |  | 850 | 85.0 | 1.9 | 86.4 | 3.2 |
